# Baseline Results: The Association Between Cardiovascular Risk and Preclinical Alzheimer’s Disease Pathology (ASCEND) Study

**DOI:** 10.3233/JAD-191103

**Published:** 2020-05-05

**Authors:** Veena V. Kumar, Hanfeng Huang, Liping Zhao, Danielle D. Verble, Alexandra Nutaitis, Sonum D. Tharwani, Alexandra L. Brown, Henrik Zetterberg, William Hu, Ryan Shin, Patrick G. Kehoe, Arshed Quyyumi, Joe Nocera, Andrea Kippels, Whitney Wharton

**Affiliations:** a Department of Neurology, Emory University School of Medicine, Atlanta, GA, USA; b Department of Biostatistics and Bioinformatics, Emory University Rollins School of Public Health, Atlanta, GA, USA; c Institute of Neuroscience and Physiology, Department Psychiatry and Neurochemistry, the Sahlgrenska Academy at the University of Gothenburg, Mölndal, Sweden; d Dementia Research Group, Faculty of Health Sciences, University of Bristol, Learning and Research, Southmead Hospital, Bristol, UK; e Division of Cardiology, Emory University School of Medicine, Atlanta, GA, USA; f Emory University School of Nursing, Atlanta, GA, USA

**Keywords:** Alzheimer’s disease, cognition, hypertension, parental history, prevention, tau, vascular risk

## Abstract

**Background:**

The rate of AD for African Americans (AAs) is 64% higher than for non-Hispanic White Americans (Whites). It is hypothesized that poor peripheral vascular function, in combination with genetics, stress, and inflammation may directly contribute to the accumulation of AD pathologic biomarkers. These risk factors may disproportionately affect AAs.

**Objective:**

Our objective was to determine if in a healthy middle-aged cohort at risk for AD (1) AD biomarkers in CSF differ by race, (2) peripheral vascular dysfunction and cognition are related to a higher burden of CSF AD biomarkers, and (3) these relationships differ by race.

**Methods:**

We enrolled 82 cognitively normal, middle-aged (45 and older) adults including AAs and Whites at high risk for AD due to parental history. Study procedures included lumbar puncture, vascular ultrasound, and cognitive testing.

**Results:**

While participants were in overall good health, AAs exhibited poorer indices of preclinical vascular health, including higher central SBP, central MAP, and EndoPAT AI, a marker of arterial stiffness. AAs also had significantly less cerebrospinal fluid tau burden than Whites. After polynomial regression analysis, adjusted for age, gender, education, and ApoE4 status, race significantly modified the relationship between total tau, phospho-tau, and Trails B, a marker of executive function. Small differences in tau correlated with poorer cognition in AAs.

**Conclusion:**

In a healthy middle-aged cohort at risk for AD, AAs had worse peripheral vascular health and worse cognition than Whites. Despite lower tau burden overall, race modified the relationship between tau and cognition, such that small differences in tau between AAs was related to worse cognition when compared to Whites.

## INTRODUCTION

With the projected increase in Alzheimer’s disease (AD), there is increased urgency to find ways of preventing or delaying disease onset. Targeting modifiable risk factors in high risk populations may help slow AD progression, though no interventions targeting these factors currently exist. Midlife vascular risk factors, particularly hypertension, have been associated with increased risk of AD in later life [1–4]. Moreover, controlling blood pressure (BP) protects against AD [2, 5, 6]; however, the mechanism of this relationship remains disputed.

The rate of AD for African Americans (AAs) is 64% higher than for non-Hispanic White Americans (Whites) [7]. The reason is likely multi-factorial and includes sociocultural issues, psychological (i.e., stress and depression), genetics, and vascular risk factors. AAs have higher cardiovascular risk including higher rates of hypertension, obesity, and diabetes [8]. The Black race is also associated with impaired microvascular vasodilatory function, and greater large arterial wave reflections and stiffness, independent of cardiovascular risk [9]. AAs report higher levels of stress and depression which increases risk of AD [10–13]. Offspring of parents with AD are six times more likely to develop AD, and the presence of family history with *APOE* *ɛ*4 positivity increases lifetime risk of AD [14].

The relationship between arterial function and cognition in healthy and AD populations has been well documented. The mechanism for this is not fully understood, but research shows midlife vascular problems may impact late life cognitive decline and AD. For example, reduced cardiac output during middle-age reduces cerebral blood flow and mediates amyloid-β (Aβ) accumulation and subsequent neuronal atrophy [15]. The Honolulu Heart Program/Honolulu-Asia Aging Study showed that midlife systolic BP variation is associated with increased Aβ in the hippocampus [16, 17]. Uncontrolled BP may be linked to Aβ accumulation in midlife, eventually leading to neuronal atrophy and cognitive impairment [18]. Studies among AD and mild cognitive impairment (MCI) patients have shown a relationship between poorer cognition and nighttime BP disruption, a common symptom among AD patients [19]. Abnormal nocturnal BP patterns and arterial stiffness are strong indicators of MCI, suggesting that arterial dysfunction may predict AD-related cognitive decline, and thus is a potential modifiable risk factor and therapeutic target for high risk individuals [20].

Here we describe the rationale, and baseline results of the **AS**sociation between **C**ardiovascular Risk and Precli**N**ical Alzheimer’s **D**isease (ASCEND) Study. ASCEND is a longitudinal, two-year observational study of cognitively normal, middle-aged adults at risk for AD, due to a parental history and overrepresentation of the ApoE *ɛ*4 allele.

The main objective of ASCEND was to determine the extent to which peripheral vascular function is related to CSF Aβ and tau levels and cognitive test performance over two years, and whether these relationships differ by race. We chose midlife because this is the time when AD neuropathology manifests, and when the negative impact of sustained vascular complications begin to have a lasting impact and arterial dysfunction begin. Importantly, midlife is the optimal time to stage an intervention via modifiable risk factors, with the potential to both reduce the likelihood of cardiovascular events and reduce the probability of AD in later life.

Herein, we report the baseline cross-sectional data comparing vascular function, cognition, AD biomarkers and race-related differences. Our hypotheses were that (1) AD biomarkers in CSF will differ by race, (2) peripheral vascular dysfunction and cognition are related to a higher burden of CSF AD biomarkers, and (3) that these relationships differ by race.

## METHODS

### ASCEND study visit and design

We enrolled eighty-two middle-aged (45 years or older) adult children of persons with AD. AD diagnosis was either autopsy-confirmed or probable AD as defined by NINDS-ADRDA criteria, and verified using the validated Dementia Questionnaire (DQ) [21] and medical records when available. Participants were recruited from the Emory Alzheimer’s Disease Research Center (ADRC) clinical cohort, physician referral, and through community events and received $100 compensation.

Table 1 shows ASCEND visit procedures at each time point. Study duration was 2 years, with annual visits (baseline, Year 1, and Year 2) although we only report baseline results in this paper. Baseline visits included a fasting lumbar puncture (LP), vascular ultrasound, ambulatory blood pressure monitoring, blood draw and cognitive testing. Study visit duration was 6.5 hours and was split into 2 days if necessary.

**Table 1:** ASCEND Study Visit Procedures

	Baseline	Year 1	Year 2
Blood Draw	X	X	X
Vascular Ultrasound (EndoPAT, FMD, PWV)	X	X	X
Ambulatory Blood Pressure	X	X	X
CSF Collection	X	–	X
Neuropsychological Testing	X	X	X

*Inclusion Criteria:* (i) a biological parent with AD; (ii) 45 – 65 years; (iii) willing to fast for eight hours; (iv) willing to undergo all procedures including LP.

*Exclusion Criteria:* (i) contraindication for LP; (ii) significant neurologic disease; (iii) history of significant head trauma; (iv) Major untreated depression within two years; (v) history of alcohol or substance abuse; (vi) any significant systemic illness or unstable medical condition which could affect cognition or cause difficulty complying with the protocol; (vii) diagnosis of AD, MCI or residence in a skilled nursing facility; (viii) use of investigational medication; (ix) unwillingness to fast.

### Procedures

*CSF collection:* Participants had CSF acquired via LP. CSF samples were collected after an 8-hour overnight fast and according to guidelines put forth in the “Biospecimens Best Practice Guidelines for the ADCs” [22]. Participants were placed in the sitting position and asked to maximally flex their knees, hips, back, and neck. The skin over L4-L5 was prepped and draped in a sterile manner. 1% lidocaine was used as a local anesthetic, followed by insertion of a spinal needle with introducer into the L4-L5 interspace using sterile technique. Approximately 22 ml of CSF was collected using sterile polypropylene collection tubes. Samples underwent a light spin and aliquoted into 500*μ*l polypropylene cryovials and stored at –80°C.

CSF t-tau concentration was determined using a sandwich ELISA (Innotest hTAU-Ag, Innogenetics, Ghent, Belgium) specifically constructed to measure all tau isoforms irrespective of phosphorylation status, as previously described [23]. Tau phosphorylated at threonine 181 (P-tau) was measured using a sandwich ELISA method (Innotest phospho-tau (181P), Innogenetics, Ghent, Belgium), as previously described [24]. Aβ_1–42_ levels were determined using a sandwich ELISA (Innotest β-Amyloid(1–42), Innogenetics, Gent, Belgium), specifically constructed to measure Aβ containing both the first and 42nd amino acid, as previously described [25]. Samples were assayed in two batches by experienced and board-certified laboratory technicians. Intra-assay coefficients of variation were below 10% for all three analytes.

*Vascular ultrasound:* Vascular measurements performed included pulse wave velocity (PWV), flow-mediated vasodilation (FMD), and pulsatile arterial tonometry (EndoPAT). Measures were chosen based on previous reports of vascular function and AD; preclinical variation in these measures may indicate vascular dysfunction before more overt clinical events occur, including hypertension or heart failure [20, 23].

PWV, a measure of arterial stiffness, is highly reproducible and correlates with MCI, cardiovascular events and all-cause mortality [20, 24]. FMD measures arterial endothelial function using high-frequency ultrasound assessment of changes in brachial artery diameter during hyperemia, and is associated with cardiovascular risk [25, 26]. EndoPAT is used to assess hyperemic microvascular function. Importantly, these markers may give us a clearer window into early vascular dysfunction in a clinically healthy population.

*PWV:* PWV was measured using an AtCor SphymoCor Px tonometry system. A pressure transducer was placed on the skin at the point the arterial pulsation of the right common carotid and right radial arteries. A Millar micromanometer was in the tip of the probe. Using a generalized transfer function, the distance between these pressure points and the peripheral arterial waveforms, a central aortic pressure signal was derived, from which aortic augmentation index and pulse wave velocity were determined.

*FMD:* Participants were placed in a supine position and an occlusion cuff was placed around the forearm. For each measurement, baseline images of the brachial artery 2–10 cm above the antecubital fossa were assessed using a 13-MHz high-resolution ultrasound transducer (Acuson). Electrocardiogram gating was used to capture end-diastolic arterial diameters and the average diameter and blood flow velocity for three cardiac cycles was recorded for baseline values. The cuff was inflated to suprasystolic levels (50 mmHg above systolic pressure) for 5 min then deflated to create a flow stimulus in the brachial artery. Doppler ultrasound was used to measure peak hyperemic blood flow velocity 10 seconds after cuff release, and diameter measurements were captured at 60 and 90 seconds after cuff release. Calculations of FMD were based on the 60 and 90 second measurements and at peak hyperemic diameters. Measurements were obtained by a trained sonographer. FMD is expressed as the % change in artery diameter from baseline: (peak hyperemic diameter – baseline diameter)/baseline diameter.

*EndoPAT:* EndoPAT detects plethysmographic pressure changes in the finger tips caused by the arterial pulse and translates this to a peripheral arterial tone. Endothelium-mediated changes in vascular tone after occlusion of the brachial artery are reflecting as downstream hyperemic response, which is a measure for arterial endothelial function. EndoPAT was measured simultaneously with FMD.

*Ambulatory blood pressure monitoring:* The National Institute for Health and Clinical Excellence (NICE) Hypertension Guidelines of the United Kingdom recommend mean 24-h ambulatory BP monitoring as a key priority in diagnosing hypertension [27]. Ambulatory measures provide a superior predictive value for cardiovascular events compared to clinic BP measurements and have been used in prior dementia research [19, 28–31].

*Blood collection:* Participants underwent blood draw for ApoE genotyping. Venous blood was collected into EDTA anticoagulated tubes and genomic DNA was isolated by standard protocols. We isolated 50 to 70 g of DNA from 2 mL of whole blood. ApoE genotypes were determined by real-time polymerase chain reaction using TaqMan probes (Applied Biosystems Inc) unique for each ApoE single-nucleotide polymorphism, rs429358 (assay ID C3084793 20) and rs7412 (assay ID C 904973 10), according to established protocols.

*Neuropsychological testing:* Neuropsychological testing lasted one hour and included tasks selectively chosen to be used in a cognitively normal but high-risk sample. The battery provided assessment of several cognitive domains (memory, executive, and visuospatial) including the Montreal Cognitive Assessment (MoCA) [32], Trail making test [33], Forwards and Backwards digit span memory test [34], Mental Rotation Test [35], Benson complex figure recall [36], Buschke memory test [37], and the Multilingual Naming Test (MINT) [38].

*Medical and medication history* including smoking history, education, income, hours of sleep, medications, history of diabetes, hypercholesterolemia, coronary artery disease, and hypertension were collected.

### Statistical methods

Demographic and clinical characteristics were summarized using descriptive statistics. Differences in categorical variables between AAs and Whites were analyzed by a chi-square test. Data normality of continuous variables was assessed by histogram and Shapiro-Wilk test. Differences for continuous variables between the two racial groups were compared with a two-sample *t*-test for normally distributed data or Mann-Whitney *U* test for variables with non-normal distribution.

The raw scores of all cognitive tests were transformed to Z scores. The resulting distribution of Z scores had a mean of 0 and a standard deviation of 1 regardless of the metric of the raw scores. Natural-logarithm or square transformation was applied to AD biomarkers to better fit normal distribution.

Polynomial multiple regressions were used to investigate associations between vascular function, AD biomarkers and cognitive test scores to detect curvilinearity in the relationship. To control the confounding factors, age, gender, race, education attainment, and ApoE *ɛ*4 status were included in the regression models. To test if the association differed by race, an interaction between each of the vascular function indices or AD biomarkers and race was included in the models. Because multiple hypotheses were tested, the false discovery rate (FDR) method was used to correct for the multiple testing problem [39].

All analyses were carried out in SAS 9.4. All tests performed were two-sided.

## RESULTS

82 individuals were enrolled. One participant was lost to follow-up, and one withdrew from the study. Of the 80 remaining individuals, 30 were AA and 50 were White (Table 2). For AAs, mean age was 60.1±7.9, 83.3% were female, and 51.7% were ApoE *ɛ*4 positive. For Whites, mean age was 58.5±6.1, 56.0% were female, and 50.0% were ApoE *ɛ*4 positive. Education level was comparable, but income was higher in Whites (*p* < 0.01). Smoking history and hypercholesterolemia were comparable between groups. More (57.1%) of AAs compared to of Whites (34.0%) had a history of hypertension (*p* = 0.0472); 40.0% of AAs compared to 20.0% Whites (*p* = 0.05) were taking antihypertensives. AAs reported 1.2 fewer hours of sleep on average (*p* < 0.01).

**Table 2:** Demographic characteristics

	African American (*n* = 30)	White (*n* = 50)	*p*
Age	60.1±7.8	58.5±6.1	0.3
Gender (% female)	83.3% *	56.00%	0.0123*
Education	10.7% High School/GED	18.0% High School/GED	0.68
	39.3% College graduate	38.0% College graduate
	50.0% Post-graduate	44.0% Post-graduate
Income	10.7% $19,000 or less*		0.0005*
	17.9% $20–39,000	12.0% $20–39,000
	28.6% $40–59,000	4.0% $40–59,000
	17.9% $60–79,000	18.0% $60–79,000
	25.0% $80,000 or more	66.0% $80,000 or more
History of smoking	30.80%	28.00%	0.8
History of diabetes	7.4% *	0%	**0.05**
History of high cholesterol	60.70%	58.00%	0.82
History of coronary artery disease	3.60%	0%	0.18
History of hypertension	57.1% *	34.00%	0.0472*
Anti-hypertensive user	40.0% *	20.00%	**0.05**
Hours of sleep	6.0±1.1*	7.2±0.9	<0.0001*
ApoE *ɛ*4 status	48.30%	50.00%	0.88

*p < 0.05.

In healthy individuals, t-tau<400, p-tau<80, Aβ_42_ _ >_ 550, and Aβ_42/40_ _ >_ 0.089. On average, participants of both races were within normal limits. However, AAs had lower t-tau and p-tau in CSF compared to Whites (*p* = 0.0036, *p* = 0.0055) (Table 3). AAs also had borderline lower Aβ_1-40_ and significantly lower Aβ_1-38_ compared to Whites (*p* = 0.07, *p* = 0.03) (Table 3).

**Table 3:** Alzheimer’s Disease Biomarkers

	African American (*n* = 30)	White (*n* = 50)	*p*
**Aβ** (pg/ml)
Triplex Aβ_1-38_	2029.2±672.9*	2466.3±760.3	0.0268*
Triplex Aβ_1-40_	5020.9±1312.7	5750.0±1618.2	0.07
Triplex Aβ_1-42_	413.1±107.5	419.9±150.0	0.85
ELISA Aβ_1-42_	722.0±164.2	703.7±197.3	0.71
**Tau** (pg/ml)			***p* (*Wilcoxen*)**
t-tau	199.0 (166.0 – 244.0)	297.0* (228.0 – 423.0)	0.0036*
p-tau	37.0 (34.0 – 42.0)	48.0* (37.0 – 64.0)	0.0055*

*p < 0.05. Aβ_1-42_/Aβ_1-40_, t-tau, and p-tau were non-parametric and necessitated use of Wilcoxen tests. Reported values for these variables are medians.

Mean peripheral BP values were similar between groups and in the pre-hypertensive range [(127.6±13.3)/(77.3±7.0) for AAs and (125.1±12.3)/(77±9.0) for Whites] with no difference in nighttime BP patterns (Table 4). FMD, PWV, RHI, and central AI were comparable between groups, but EndoPAT AI (*p* = 0.01) and central BP (*p* = 0.017) were significantly higher in AAs compared to Whites, with central pressure trending higher in AAs (*p* = 0.06).

**Table 4:** Vascular risk factors

	African American (*n* = 30)	White (*n* = 50)	*p*
**Ambulatory Blood Pressure**
Systolic (mmHg)	127.6±13.3	125.1±12.3	0.42
Diastolic (mmHg)	77.3±7.0	77.3±9.0	0.99
**Endothelial Function**
FMD (%)	6.4±5.2	5.3±4.5	0.33
VTI (cm)	7.3±2.9	6.2±2.4	0.10
Peak velocity (cm/s)	1.1±0.6	1.1±0.4	0.59
EndoPAT-RHI	2.3±0.7	2.3±0.7	0.89
EndoPAT-AI (%)	33.9±19.9*	22.0±17.1	0.0102*
PWV (m/s)	7.6±1.5	7.3±1.3	0.36
Central Systolic (mmHg)	121.2±19.5*	110.8±12.5	0.0174*
Central Diastolic (mmHg)	79.1±13.0	75.1±10.7	0.16
Central MAP units (mmHg)	96.5±14.7	90.3±10.4	**0.06**
Central AI (%)	150.2±22.3	143.3±20.7	0.189

*p < 0.05.

AAs performed more poorly on all cognitive tests compared to Whites, with significant differences in the MoCA, Trails B, Buschke delay, and MINT tests (*p* < 0.01, *p* = 0.02, *p* = 0.02, *p* < 0.01) (Table 5).

**Table 5:** Cognitive testing

	African American (*n* = 30)	White (*n* = 50)	*p* (Wilcoxen)
MoCA	25.0 (24.0 – 27.0)*	27.0 (25.0 – 29.0)	0.0051*
Trails B	81.0 (69.0 – 101)*	70.0 (56.0 – 82.0)	0.0239*
Forwards Digit Span	6.5 (5.5 – 7.0)	7.0 (6.0 – 8.0)	0.22
Backwards Digit Span	4.0 (3.0 – 5.0)	5.0 (4.0 – 6.0)	0.10
Mental Rotation	17.5 (15.0 – 20.0)	18.5 (13.0 – 21.0)	0.50
Benson Delay	12.0 (10.0 – 14.0)	12.0 (10.0 – 13.0)	0.28
Buschke Delay	6.0 (2.0 – 8.0)*	7.0 (5.0 – 9.0)	0.0218*
MINT	29.0 (28.0 – 31.0)*	31.0 (30.0 – 32.0)	0.0017*

*p < 0.05. Reported values are medians.

**Fig.1:**
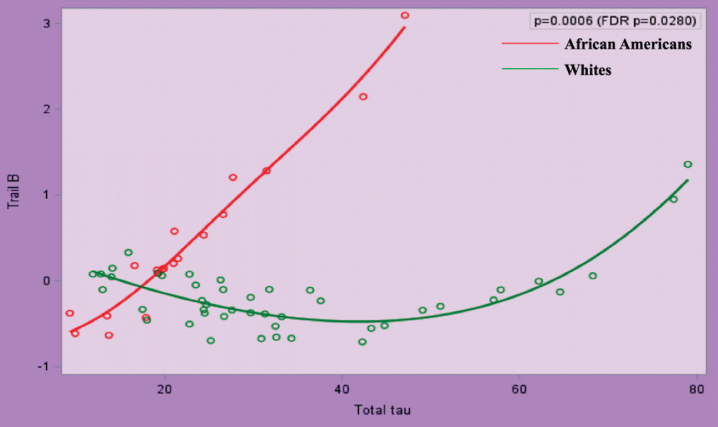
Relationship between Trail B and Total Tau in African Americans and Whites Adjusted for Age, Gender, Education Attainment, and ApoE4 Status.

**Fig.2:**
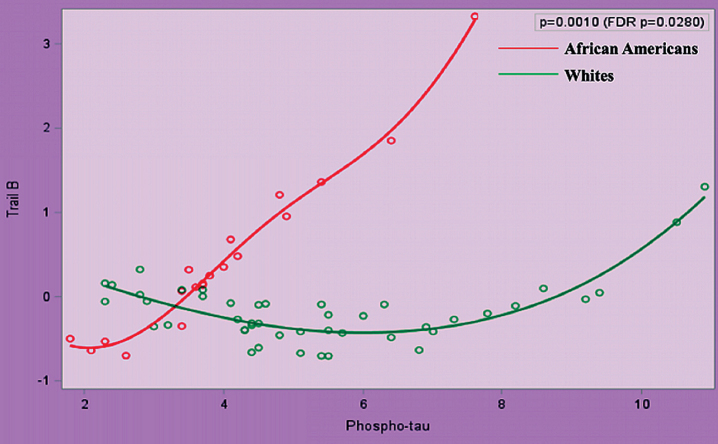
Relationship between Trail B and Phospho-Tau in African Americans and Whites Adjusted for Age, Gender, Education Attainment, and ApoE4 Status.

After transformations for non-normally distributed data, we performed polynomial regression analyses between AD biomarkers and (a) cognition, (b) peripheral vascular function, and (c) between peripheral vascular function and cognition. Race significantly modified the relationship between Z score of Trail B and t-tau and p-tau (false discovery rate *p* = 0.0280 for both), after adjustment for age, gender, education, and ApoE *ɛ*4 (Figs. 1 and 2).

## DISCUSSION

In this study, we present baseline data of the ASCEND study, a sampling of 30 AA and 50 White, asymptomatic and middle-aged individuals at high-risk for AD due to parental history. Women were more proportionately represented in the AA group, and incomes were higher in the White cohort despite similar education levels. More AAs reported hypertension and antihypertensive medication use, coronary artery disease, and diabetes, which is consistent with prior research showing poorer cardiovascular health among AAs [8]. Approximately half of the participants in both racial groups were ApoE *ɛ*4 positive, consistent with our and other studies in cohorts of adult children of AD individuals [6, 40].

Whites outperformed AAs on all cognitive tests, including global cognition, executive function, and verbal memory and language. Cognitive tests may have implicit cultural biases which favor Whites [41, 42]. Depression and stress, which result in poorer cognitive test performance, have higher incidences in AAs [43]; however, the incidence of both in our cohort did not differ by race. AAs did report less sleep than their White peers. Low and high blood pressures can also result in poorer performance; however, our lowest recorded BP was 104/59 and the majority were in the normal or pre-hypertensive range.

AAs had lower levels of tau (t-tau, p-tau) compared to Whites. This result was first reported in 2017 in older adults and proved true both in cognitively normal and cognitively impaired groups [40]. The finding was corroborated in 2019 in a larger population of adults which also included both cognitively normal and impaired individuals [44]. Our study is the first to report this in a cohort of middle-aged, cognitively normal adults at risk for AD. The fact that this discrepancy is present even during middle-age supports the hypothesis that AD neuropathology begins during middle-age before the clinical or cognitive symptoms of AD manifest. It is possible that race related differences in brain AD biomarkers exist. Further research is needed to ensure the appropriateness of tau and Aβ targeted therapies in both AAs and Whites, or whether modifications should be made based on race.

Race significantly modified the relationship between tau (t-tau and p-tau) and executive function. Executive function is one of the first cognitive domains affected in AD, and tau has been correlated to MCI. It has previously been postulated that despite lower baseline levels of tau, AAs are sensitive to smaller changes in tau as evidenced by worse cognition, similar hippocampal atrophy, and white matter hyperintensity [42]. Our results are cross-sectional and therefore we cannot report whether individuals are more sensitive to smaller changes in tau; however, as a group, small differences in tau in AAs were related to worse cognition when compared to Whites. Morris et al. found a significant race by ApoE *ɛ*4 interaction for tau such that only *ɛ*4 positive participants showed racial differences [41]. Our cohort was strongly ApoE *ɛ*4 positive, yet even after adjusting for this we found the correlation between tau and Trails B to hold true. The finding that race modifies the relationship between tau and cognition implies that the neuropathology of tau deposition may differ in AAs, and there may need to be a lower threshold for treatment in these individuals. Existing cutoff values for CSF biomarkers may not be appropriate for AAs.

We report that AAs have lower levels of Aβ_38_. Both Aβ_40_ and Aβ_38_ variants are more abundant in the brain than Aβ_1-42_. Future studies should investigate the role of these lesser studied forms of Aβ in AD pathology.

While BP values were not different in AAs and Whites in our study, AAs self-reported more hypertension, antihypertensive medications,and diabetes. However, BP was similar and in the pre-hypertensive range for both AAs and Whites, which makes the finding of microvascular differences still significant. A previous study of racial difference in microvascular health found that AAs had greater arterial wave reflections as assessed by EndoPAT AI and central AI, and greater arterial stiffness as assessed by PWV, even after adjustment for cardiovascular risk factors [9]. Our results for EndoPAT AI are similar although central AI and PWV differences were not significantly different in our smaller cohort, probably due to the smaller sample size. EndoPAT is a marker of arterial stiffness and has been shown to be related to white matter microstructure and executive function [43]. This is important because in our middle-aged cohort higher EndoPAT, i.e., stiffer arteries may be a precursor to possible cognitive decline. There was no significant relationship between EndoPAT and cognition in our baseline results, but it would be interesting to watch this relationship over the course of two years, or in future studies even longer. We found AAs had higher central systolic BP and central mean arterial pressures, as measured by pulse wave analysis. Central BP is generally  10 mmHg lower than the peripheral BP, which was true in the White participants [44]. Interestingly, in the AA group, central BP was similar to peripheral BP and higher than in Whites. Pressures within the central aorta may be more relevant to cardiovascular outcome than pressures in the brachial artery, although its clinical relevance remains controversial [44, 45]. It is possible that aortic BP, rather than peripheral BP, might be an early mid-life difference of vascular risk for AAs specifically, even before development of clinical hypertension.

## References

[1] Bendlin BB , Carlsson CM , Gleason CE , Johnson SC , Sodhi A , Gallagher CL , Puglielli L , Engelman CD , Ries ML , Xu G , Wharton W , Asthana S (2010) Midlife predictors of Alzheimer’s disease. Maturitas 65, 131–137. 20044221 10.1016/j.maturitas.2009.12.014 PMC2895971

[2] Goldstein FC , Ashley AV , Freedman LJ , Penix L , Lah JJ , Hanfelt J , Levey AI (2005) Hypertension and cognitive performance in African Americans with Alzheimer disease. Neurology 64, 899–901. 15753433 10.1212/01.WNL.0000152888.26576.37

[3] Kivipelto M , Helkala EL , Hanninen T , Laakso MP , Hallikainen M , Alhainen K , Soininen H , Tuomilehto J , Nissinen A (2001) Midlife vascular risk factors and late-life mild cognitive impairment: A population-based study. Neurology 56, 1683–1689. 11425934 10.1212/wnl.56.12.1683

[4] Kivipelto M , Helkala EL , Laakso MP , Hanninen T , Hallikainen M , Alhainen K , Soininen H , Tuomilehto J , Nissinen A (2001) Midlife vascular risk factors and Alzheimer’s disease in later life: Longitudinal, population based study. BMJ 322, 1447–1451. 11408299 10.1136/bmj.322.7300.1447 PMC32306

[5] Wharton W , Goldstein FC , Zhao L , Steenland K , Levey AI , Hajjar I (2015) Modulation of renin-angiotensin system may slow conversion from mild cognitive impairment to Alzheimer’s disease. J Am Geriatr Soc 63, 1749–1756. 26389987 10.1111/jgs.13627 PMC4743657

[6] Wharton W , Stein JH , Korcarz C , Sachs J , Olson SR , Zetterberg H , Dowling M , Ye S , Gleason CE , Underbakke G , Jacobson LE , Johnson SC , Sager MA , Asthana S , Carlsson CM (2012) The effects of ramipril in individuals at risk for Alzheimer’s disease: Results of a pilot clinical trial. J Alzheimers Dis 32, 147–156. 22776970 10.3233/JAD-2012-120763 PMC3593582

[7] Steenland K , Goldstein FC , Levey A , Wharton W (2015) A meta-analysis of Alzheimer’s disease incidence and prevalence comparing African-Americans and Caucasians. J Alzheimers Dis 50, 71–76. 10.3233/JAD-150778 PMC4739787 26639973

[8] Gu Q , Burt VL , Paulose-Ram R , Yoon S , Gillum RF (2008) High blood pressure and cardiovascular disease mortality risk among U.S. adults: The third National Health and Nutrition Examination Survey mortality follow-up study. Ann Epidemiol 18, 302–309. 18261929 10.1016/j.annepidem.2007.11.013

[9] Morris AA , Patel RS , Binongo JN , Poole J , Al Mheid I , Ahmed Y , Stoyanova N , Vaccarino V , Din-Dzietham R , Gibbons GH , Quyyumi A (2013) Racial differences in arterial stiffness and microcirculatory function between Black and White Americans. J Am Heart Assoc 2, e002154. 23568343 10.1161/JAHA.112.002154 PMC3647269

[10] Machado A , Herrera AJ , de Pablos RM , Espinosa-Oliva AM , Sarmiento M , Ayala A , Venero JL , Santiago M , Villarán RF , Delgado-Cortés MJ , Argüelles S , Cano J (2014) Chronic stress as a risk factor for Alzheimer’s disease. Rev Neurosci 25, 785–804. 25178904 10.1515/revneuro-2014-0035

[11] Williams DR , González HM , Neighbors H , Nesse R , Abelson JM , Sweetman J , Jackson JS (2007) Prevalence and distribution of major depressive disorder in African Americans, Caribbean blacks, and non-Hispanic whites: Results from the National Survey of American Life. Arch Gen Psychiatry 64, 305–315. 17339519 10.1001/archpsyc.64.3.305

[12] Clark R , Anderson NB , Clark VR , Williams DR (1999) Racism as a stressor for African Americans. A biopsychosocial model. Am Psychol 54, 805–816. 10540593 10.1037//0003-066x.54.10.805

[13] Evans DA , Bennett DA , Wilson RS , Bienias JL , Morris MC , Scherr PA , Hebert LE , Aggarwal N , Beckett LA , Joglekar R , Berry-Kravis E , Schneider J (2003) Incidence of Alzheimer disease in a biracial urban community: Relation to apolipoprotein E allele status. Arch Neurol 60, 185–189. 12580702 10.1001/archneur.60.2.185

[14] Green RC , Cupples LA , Go R , Benke KS , Edeki T , Griffith PA , Williams M , Hipps Y , Graff-Radford N , Bachman D , Farrer LA (2002) Risk of dementia among white and African American relatives of patients with Alzheimer disease. JAMA 287, 329–336. 11790212 10.1001/jama.287.3.329

[15] Jefferson AL , Poppas A , Paul RH , Cohen RA (2007) Systemic hypoperfusion is associated with executive dysfunction in geriatric cardiac patients. Neurobiol Aging 28, 477–483. 16469418 10.1016/j.neurobiolaging.2006.01.001 PMC2741683

[16] Freitag MH , Peila R , Masaki K , Petrovitch H , Ross GW , White LR , Launer LJ (2006) Midlife pulse pressure and incidence of dementia: The Honolulu-Asia Aging Study. Stroke 37, 33–37. 16339468 10.1161/01.STR.0000196941.58869.2d

[17] Launer LJ , Ross GW , Petrovitch H , Masaki K , Foley D , White LR , Havlik RJ (2000) Midlife blood pressure and dementia: The Honolulu-Asia aging study. Neurobiol Aging 21, 49–55. 10794848 10.1016/s0197-4580(00)00096-8

[18] Petrovitch H , White LR , Izmirilian G , Ross GW , Havlik RJ , Markesbery W , Nelson J , Davis DG , Hardman J , Foley DJ , Launer LJ (2000) Midlife blood pressure and neuritic plaques, neurofibrillary tangles, and brain weight at death: The HAAS. Honolulu-Asia aging Study. Neurobiol Aging 21, 57–62. 10794849 10.1016/s0197-4580(00)00106-8

[19] Guo H, Tabara Y, Igase M, Yamamoto M, Ochi N, Kido T, Uetani E, Taguchi K, Miki T, Kohara K Abnormal nocturnal blood pressure profile is associated with mild cognitive impairment in the elderly: The J-SHIPP study. Hypertens Res 33, 32–36. 19851324 10.1038/hr.2009.172

[20] Hanon O , Haulon S , Lenoir H , Seux ML , Rigaud AS , Safar M , Girerd X , Forette F (2005) Relationship between arterial stiffness and cognitive function in elderly subjects with complaints of memory loss. Stroke 36, 2193–2197. 16151027 10.1161/01.STR.0000181771.82518.1c

[21] Kawas C , Segal J , Stewart WF , Corrada M , Thal LJ (1994) A validation study of the Dementia Questionnaire. Arch Neurol 51, 901–906. 8080390 10.1001/archneur.1994.00540210073015

[22] National Institute on Aging (2014), *Biospecimen best practice guidelines for the Alzheimer’s Disease Centers*. https://www.alz.washington.edu/BiospecimenTaskForce.html.

[23] Hajjar I , Goldstein FC , Martin GS , Quyyumi AA (2016) Roles of arterial stiffness and blood pressure in hypertension-associated cognitive decline in healthy adults. Hypertension 67, 171–175. 26527049 10.1161/HYPERTENSIONAHA.115.06277 PMC4715367

[24] Blacher J , Asmar R , Djane S , London GM , Safar ME (1999) Aortic pulse wave velocity as a marker of cardiovascular risk in hypertensive patients. Hypertension 33, 1111–1117. 10334796 10.1161/01.hyp.33.5.1111

[25] Corretti MC , Anderson TJ , Benjamin EJ , Celermajer D , Charbonneau F , Creager MA , Deanfield J , Drexler H , Gerhard-Herman M , Herrington D , Vallance P , Vita J , Vogel R (2002) Guidelines for the ultrasound assessment of endothelial-dependent flow-mediated vasodilation of the brachial artery: A report of the International Brachial Artery Reactivity Task Force. J Am Coll Cardiol 39, 257–265. 11788217 10.1016/s0735-1097(01)01746-6

[26] Kuvin JT , Patel AR , Sliney KA , Pandian NG , Sheffy J , Schnall RP , Karas RH , Udelson JE (2003) Assessment of peripheral vascular endothelial function with finger arterial pulse wave amplitude. Am Heart J 146, 168–174. 12851627 10.1016/S0002-8703(03)00094-2

[27] McCormack T , Krause T , O’Flynn N (2012) Management of hypertension in adults in primary care: NICE guideline. Br J Gen Pract 62, 163–164. 22429432 10.3399/bjgp12X630232 PMC3289819

[28] Burnier M, Grassi G Ambulatory blood pressure monitoring in diabetic patients: New data, new questions. J Hypertens 29, 198–200. 21191278 10.1097/HJH.0b013e328342d4d7

[29] Nagai M , Hoshide S , Ishikawa J , Shimada K , Kario K (2008) Ambulatory blood pressure as an independent determinant of brain atrophy and cognitive function in elderly hypertension. J Hypertens 26, 1636–1641. 18622243 10.1097/HJH.0b013e3283018333

[30] Pickering TG (2000) Ambulatory blood pressure monitoring. Curr Hypertens Rep 2, 558–564. 11062602 10.1007/s11906-996-0041-8

[31] van Boxtel MP , Henskens LH , Kroon AA , Hofman PA , Gronenschild EH , Jolles J , de Leeuw PW (2006) Ambulatory blood pressure, asymptomatic cerebrovascular damage and cognitive function in essential hypertension. J Hum Hypertens 20, 5–13. 16163365 10.1038/sj.jhh.1001934

[32] Nasreddine ZS , Phillips NA , Bedirian V , Charbonneau S , Whitehead V , Collin I , Cummings JL , Chertkow H (2005) The Montreal Cognitive Assessment, MoCA: A brief screening tool for mild cognitive impairment. J Am Geriatr Soc 53, 695–699. 15817019 10.1111/j.1532-5415.2005.53221.x

[33] Bowie CR , Harvey PD (2006) Administration and interpretation of the Trail Making Test. Nat Protoc 1, 2277–2281. 17406468 10.1038/nprot.2006.390

[34] Wechsler D (1991) WAIS-R Wechsler adult intelligence scale-III, Psychological Corporation, New York, N.Y.

[35] Vandenberg SG , Kuse AR (1978) Mental rotations, a group test of three-dimensional spatial visualization. Percept Mot Skills 47, 599–604. 724398 10.2466/pms.1978.47.2.599

[36] Rey A (1941) Psychological examination of traumatic encephalopathy. Archives de Psychologie 28, 286-340; sections translated by Corwin J and Blysma FW. Clin Neuropsychol 7, 4–9.

[37] Buschke H (1973) Selective reminding for analysis of memory and learning. J Verb Learn Verb Behav 12, 543–550.

[38] Ivanova I , Salmon DP , Gollan TH (2013) The multilingual naming test in Alzheimer’s disease: Clues to the origin of naming impairments. J Int Neuropsychol Soc 19, 272–283. 23298442 10.1017/S1355617712001282 PMC4356120

[39] Benjamini Y (1995) Controlling the false discovery rate: A practical and powerful approach to multiple testing. J Royal Stat Soc Ser B 57, 289–300.

[40] Aschenbrenner AJ , Balota DA , Fagan AM , Duchek JM , Benzinger TL , Morris JC (2015) Alzheimer disease cerebrospinal fluid biomarkers moderate baseline differences and predict longitudinal change in attentional control and episodic memory composites in the Adult Children Study. J Int Neuropsychol Soc 21, 573–583. 26416094 10.1017/S1355617715000776 PMC4610253

[41] Brickman AM , Cabo R , Manly JJ (2006) Ethical issues in cross-cultural neuropsychology. Appl Neuropsychol 13, 91–100. 17009882 10.1207/s15324826an1302_4

[42] Fyffe DC , Mukherjee S , Barnes LL , Manly JJ , Bennett DA , Crane PK (2011) Explaining differences in episodic memory performance among older African Americans and Whites: The roles of factors related to cognitive reserve and test bias. J Int Neuropsychol Soc 17, 625–638. 23131601 10.1017/S1355617711000476 PMC3496282

[43] Blazer DG , Kessler RC , McGonagle KA , Swartz MS (1994) The prevalence and distribution of major depression in a national community sample: The National Comorbidity Survey. Am J Psychiatry 151, 979–986. 8010383 10.1176/ajp.151.7.979

[44] Morris JC , Schindler SE , McCue LM , Moulder KL , Benzinger TLS , Cruchaga C , Fagan AM , Grant E , Gordon BA , Holtzman DM , Xiong C (2019) Assessment of racial disparities in biomarkers for Alzheimer disease. JAMA Neurol 76, 264–273. 30615028 10.1001/jamaneurol.2018.4249 PMC6439726

[45] Howell JC , Watts KD , Parker MW , Wu J , Kollhoff A , Wingo TS , Dorbin CD , Qiu D , Hu WT (2017) Race modifies the relationship between cognition and Alzheimer’s disease cerebrospinal fluid biomarkers. Alzheimers Res Ther 9, 88. 29096697 10.1186/s13195-017-0315-1 PMC5668981

[46] Johnson NF , Gold BT , Brown CA , Anggelis EF , Bailey AL , Clasey JL , Powell DK (2017) Endothelial function is associated with white matter microstructure and executive function in older adults. Front Aging Neurosci 9, 255. 28824417 10.3389/fnagi.2017.00255 PMC5539079

[47] Williams B , Lacy PS , Thom SM , Cruickshank K , Stanton A , Collier D , Hughes AD , Thurston H , O’Rourke M , CAFE Investigators; Anglo-Scandinavian Cardiac Outcomes Trial Investigators; CAFE Steering Committee and Writing Committee (2006) Differential impact of blood pressure-lowering drugs on central aortic pressure and clinical outcomes: Principal results of the Conduit Artery Function Evaluation (CAFE) study. Circulation 113, 1213–1225. 16476843 10.1161/CIRCULATIONAHA.105.595496

[48] London G , Guerin A , Pannier B , Marchais S , Benetos A , Safar M (1992) Increased systolic pressure in chronic uremia. Role of arterial wave reflections. Hypertension 20, 10–19. 1618545 10.1161/01.hyp.20.1.10

